# Short Term Preservation of Hide Using Vacuum: Influence on Properties of Hide and of Processed Leather

**DOI:** 10.1371/journal.pone.0112783

**Published:** 2014-11-13

**Authors:** Ilze Gudro, Virgilijus Valeika, Justa Sirvaitytė

**Affiliations:** 1 Textile Material Technology and Design Institute, Riga Technical University, Riga, Latvia; 2 Department of Physical and Inorganic Chemistry, Kaunas University of Technology, Kaunas, Lithuania; 3 Department of Polymer Chemistry and Technology, Kaunas University of Technology, Kaunas, Lithuania; University of Crete, Greece

## Abstract

The objective of this work was to investigate vacuum influence on hide preservation time and how it affects hide structure. It was established that vacuum prolongs the storage time without hide tissue putrefaction up to 21 days when the storage temperature is 4°C. The microorganisms act for all storage times, but the action is weak and has no observable influence on the quality of hide during the time period mentioned. The hide shrinkage temperature decrease is negligible, which shows that breaking of intermolecular bonds does not occur. Optical microscopy, infrared spectroscopy and differential scanning calorimetry also did not show any structural changes which can influence the quality of leather produced from such hide. The qualitative indexes of wet blue processed under laboratory conditions and of leather produced during industrial trials are presented. Indexes such as chromium compounds exhaustion, content of chromium in leather, content of soluble matter in dichloromethane, strength properties, and shrinkage temperature were determined. Properties of the leather produced from vacuumed hide under industrial conditions conformed to the requirements of shoe upper leather.

## Introduction

The leather industry is one of the oldest industries in the world, which nowadays plays a large role in the economic system worldwide. Looking through the ages, we can state that leather is a sustainable material, because while people eat meat, they will have this raw material available. Despite incomes, the leather industry is a high pollutant industry. The reason for this is that leather is not a “friend” of the environment, as it plays a role in the environmental destruction caused by the meat industry as well as the pollution caused by the materials used in leather preservation and processing. For this reason, raw hide preservation has always been a challenge for leather manufacturers.

The raw hides and skins are flayed from the animal and further processed into leather. As the main constituent of raw skins and hides is protein, these materials are highly susceptible to bacterial action. Deterioration of the skin starts within 5–6 h after flaying; hence, there is a requirement for an effective preservative. Thus, it is essential to preserve the protein matrix and also to temporarily arrest microbial attacks [1].

The preservation of raw stock has the objective of rendering the flayed skin/hide resistant to putrefaction in order to allow transport and storage. Preservation is accomplished either by destroying active bacteria, by preventing bacterial activity or by preventing bacterial contamination [2]. During preservation, it is essential to avoid the use of toxic materials, as these are very dangerous for the environment due to their chemical nature.

From ancient times, common salt (NaCl) and its sub-empirical forms like Khari salts have been employed for preservation. These salts are available to flayers and slaughterhouses globally [3].

Preservation using salt remains the most popular curing technique worldwide due to its ease, cost-effectiveness and the quality of the finished leather produced. The use of salt enhances the pollution load of tannery effluent, however, which becomes highly contaminated with increased total dissolved solids (TDS) and chlorides (Cl^–^) [4]. To overcome this hurdle, researchers are constantly searching for alternative preservation techniques which are either totally void of salt or use only a small amount.

Sundar and Muralidharan developed a low salt-MgO substituted skin preservation methodology. The methodology bases the amount of salt required on the weight of the skin to be treated, and uses less than 25% of the weight of the skin; it is also suitable for all conventional raw material resources [5]. Kuttalam et al. proposed using *Semecarpus nacardium* nut extract as an alternative to salt for the curing process by assessing different parameters like hair slip, putrefaction odour, volatile nitrogen content, moisture content, bacterial count and shrinkage temperature in comparison to the salt curing method [6].

A study of buffalo hide preservation using sodium sulphate was carried out. After rigorous laboratory experimentation on moisture content, SEM of hide, pure sodium sulphate as well as sodium sulphate in addition to sodium chloride (i.e. 10% w/w and 20% w/w) proved to be the most preferable option for curing of buffalo hide, as it gave effective preservation [7].

Materials such as polyethylene glycol [8], boric acid [9], collagenase inhibitors [10], ozone [11], and mixtures of acetic acid with sodium sulphite [12] or with benzoic acid [13] were investigated as preservatives for hides and skins.

Attempts were made to completely avoid chemicals for that purpose. Here, the use of electron beam irradiation [14] or gamma radiation [15] should be mentioned. Authors [14] have reported that a total of three weeks elapsed between hide removal and tanning. After storage at room temperature, all of the hides appeared to be as fresh and clean as they were immediately after flaying. With the exception of draw, which was considerably better on the irradiated sides compared to the brine sides, all of the parameters measured were equivalent for both preservation treatments.

Vankar and Dhivehi [3] proposed that commercial scarcity and the cost of consumption of the above-mentioned curing processes do not invite flayers to follow these new methods. These suppliers who supply 60–70% of raw skins for total consumption to the leather industry require a cheap strategy. Hence, the development of an alternative curing technique should proceed in such a way that the practices and socioeconomic feasibility can be maintained at equilibrium.

It is known that lowering the temperature decreases the pace of bacterial growth [16]. The rate of collagen and non-collagenous protein degradation and microbial population dynamics were found to vary with temperature and storage periods. Of course, hide/skin can be frozen, and then stored sufficiently for a long time [17], but the structure of hide/skin under freezing conditions changes undesirably due to the formation of ice crystals.

One of the alternative methods could be storage of hide in vacuum under low temperatures, as used for certain foods. This method could help to avoid pollution with salt and buried raw hides. On the other hand, vacuum preservation can only be applied in practice after thorough exploration of the method. The vacuum is widely used in leather processing for leather drying, as it offers fast speed at a low temperature, which would be advantageous for heat-vulnerable chrome-free leather [18]. Several attempts have been made to use vacuum for other leather processing steps, including leather tanning. The results acquired results show that, on the premise of increasing the overall quality of leather tannage, the working time is greatly shortened and the discharge of waste liquid is decreased by more than 50% [19]. The use of 0.01 MPa vacuum reduces the duration of chroming down to 3–20 minutes, depending on the thickness of the pelt [20].

The main aim of the research was to evaluate vacuum influence on hide storage time, structural changes and the properties of leather produced from hide preserved using a vacuum.

## Experiments

### Materials

**Figure 1:**
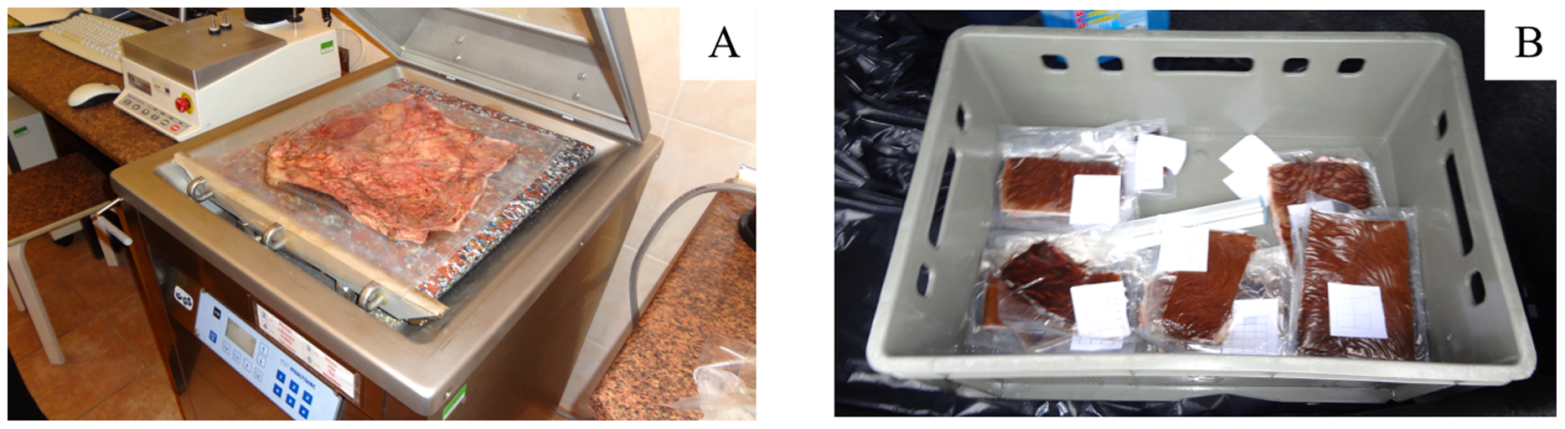
Equipment for fresh hide packing using vacuum (A) and the packed hide samples (B).

Two fresh calf hides were purchased from the local slaughterhouse (“AGARAS” Lithuania), and were cut into pieces immediately after flaying. Test pieces for further processing and analysis were taken as standard [21]. The *little* samples (10–15×20 cm) were prepared for laboratory investigations from one hide and *bigger* samples (42×36 cm) were prepared from a second hide for testing under industrial conditions. All samples (except samples for laboratory tests) were placed into special packing film using a vacuum (10–12·10^3^ Pa) (**Fig. 1**). Those hide samples entitled “**vacuumed**” were stored in a fridge at 4°C.

Four small pieces were enveloped into simple polyethylene film and stored in a fridge at 4°C (“**not preserved**” samples).

Four small pieces were salted using 50% NaCl and stored at 20±1°C (“**salted**” samples).

All not preserved, salted and four small vacuumed pieces were prepared from lower part of hide. Processing under laboratory conditions and determination of hide and leather properties were carried out using these pieces.

All chemicals used for analysis were of analytical grade. For leather processing under laboratory conditions, the chemicals used were also of analytical grade. Together, the following technical products were used: enzyme preparation *Oropon ON2* (TFL, Switzerland), basic chromium extract *Chromeco* (Dalton, Italy), and *Neutragene MG-120* (Codyeco s.p.a., Italy) for increasing the chromium compounds’ basicity.

### Methods

#### Evaluation of preservation quality

The quality of preservation was assessed organoleptically by observing any hair slip, the appearance of bad odour and the appearance of the mucous surface of the skin. The second method for preservation quality assessment was the evaluation of bacteria on the hide. A method that had been previously described in the literature was used [13]. The hide was considered as deteriorated when any symptom of decay appeared: hair slip, bad odour, or mucous surface of the skin, or when the amount of microorganisms in the skin exceeded 20×10^6^ units per 1 g of hide.

#### Determination of content of nitrogen extracted from hide

A weighed piece of hide (about 5 g) was cut into small pieces (3×3 mm). These pieces were placed into a glass flask and covered with 25 ml of distilled water. Each flask was embedded into a shaker and stirred for 30 minutes. The acquired liquid was then filtered and 10 ml of the filtrate was used for nitrogen determination according to the Kjeldahl procedure.

#### Processing of samples under laboratory and industrial conditions

A piece of the little samples was processed into wet blue under laboratory conditions, as described in Table 1. The processing was performed in laboratory drums with a capacity of 3 litres.

**Table 1:** Parameters of hide processing in laboratory conditions.

Process title	Process parameters		
	Material and amount (% based onhide/unhaired hide mass)	Temperature, °C	Duration and regimen
**Washing**	H_2_O –200	18–22	1 h, run continuously
**Liming-** **unhairing**	**a)** H_2_O –100;PAM –0.1	18–22	**a)** 30 min., run continuously
	**b)** Ca(OH)_2_ (60%) –2.3; Na_2_S (60%) –2		**b)** 1 h, run continuously
	**c)** Ca(OH)_2_ (60%) –2.3		**c)** 1 h, run continuously
	**d)** H_2_O –100		**d)** 21 h, 30 min run continuously, later 5 min. every 4 h, drain in the process end
**Washing**	**a)** H_2_O –200	37±1	**a)** 30 min., run continuously, drain
	**b)** H_2_O –200		**b)** 30 min., run continuously, drain
**Deliming-** **bating**	**a)** H_2_O –40;(NH_4_)_2_SO_4-_2	37±1	**a)** 30 min., run continuously
	**b)** (NH_4_)_2_SO_4-_2		**b)** 30 min., run continuously
	**c)** H_2_O –100; Enzyme *Oropon ON2-*0.15		**c)** 1 h, run continuously, drain
**Washing**	**a)** H_2_O –200	20–22	**a)** 20 min., run continuously, drain
	**b)** H_2_O –200		**b)** 20 min., run continuously, drain
**Pickling**	**a)** H_2_O –40; NaCl –5.5	20–22	**a)** 15 min., run continuously
	**b)** HCOONa –1		**b)** 20 min., run continuously
	**c)** H_2_SO_4-_0.5		**c)** 15 min., run continuously
	**d)** H_2_SO_4-_0.5		**d)** 15 min., run continuously
	**e)** H_2_SO_4-_0.5		**e)** 5 h, run continuously
**Chroming**	**a)** *Chromeco* –6	20–22	**a)** 20 h, run continuously
	**b)** *Neutragene MG-120-*0.35		**b)** 2 h, run continuously
	**c)** H_2_O –100	40±2	**c)** 2 h, run continuously, drain
**Washing**	H_2_O –100	40±2	1 h, run continuously, drain

Big samples were processed into wet blue under industrial conditions in the “Kedainiu oda” tannery (Lithuania) according to the technology of upper leather processing, which was valid in this enterprise.

#### Determination of hide and leather properties

During the hide sample processing under laboratory conditions, the solutions were analysed after each process. The amount of collagen protein (CP) was estimated from the amount of hydroxyproline in the solution, and the amount of hydroxyproline was determined using a photo colorimetric method [22]. Shrinkage temperature of hide samples was determined as standard [23].

Chromium compound exhaustion was established by determining the concentration of chromium in the initial chroming solution and in a mixture of used chroming solution and post-wash chroming solutions. The concentration of chromium in solution was determined according to the method described in the literature [24]. The method prescribes oxidation of the chromium presented in the solution into hexavalent state using hydrogen peroxide, and analysis of the solution by iodometric titration.

Strength properties, the amount of chrome compounds in leather, soluble matter in dichloromethane, and volatile matter were determined according to standards [25], [26], [27], [28].

Shrinkage temperature of chromed leather samples (wet blue) was determined as described in the literature [24] using special equipment and replacing the distilled water with glycerol.

#### Optical microscopy, infrared spectroscopy and differential scanning calorimetry

The optical microscopy investigation was performed with an *Olympus CX 31* microscope. An infrared (FTIR) absorption spectrum was obtained using a Perkin-Elmer FTIR Spectrum GX (USA) spectrometer using KBr pellets. The resolution was 1 cm^–1^, scan rate was 0.2 cm/s and scan number was 16. The software “Spectrum 5.0.1″ was used by calculating the area of peaks in spectra Δ*S* (T%·cm^–1^). Differential scanning calorimetry (DSC) analysis was performed with the Netzsch Gerätebau GmbH (Germany) thermal analyser in a nitrogen atmosphere with a heating rate of −10°C/min.

The hide samples for optical microscopy, FTIR spectroscopy and DSC analysis were prepared by dehydration with acetone [29].

#### Statistical analysis

All data were expressed as the average value of triplicate measurements. Confidence limits were set at P<0.05. Standard deviations did not exceed 5% for the values obtained.

## Results and Discussion

The hides and skins without any preservative enable the growth of microorganisms instantaneously, which can reach a level of several million per millilitre and can damage the hides within a period as short as 5–6 h. The predominant bacteria found in soaking baths are *Enterobacter aerogenes*, *Bacillus mycoides*, *Escherichia coli*, *Pseudomonas aeruginosa*, *Proteus vulgaris*, *Staphylococcus aureus* and other gelatine-liquefying bacteria [30].

**Figure 2:**
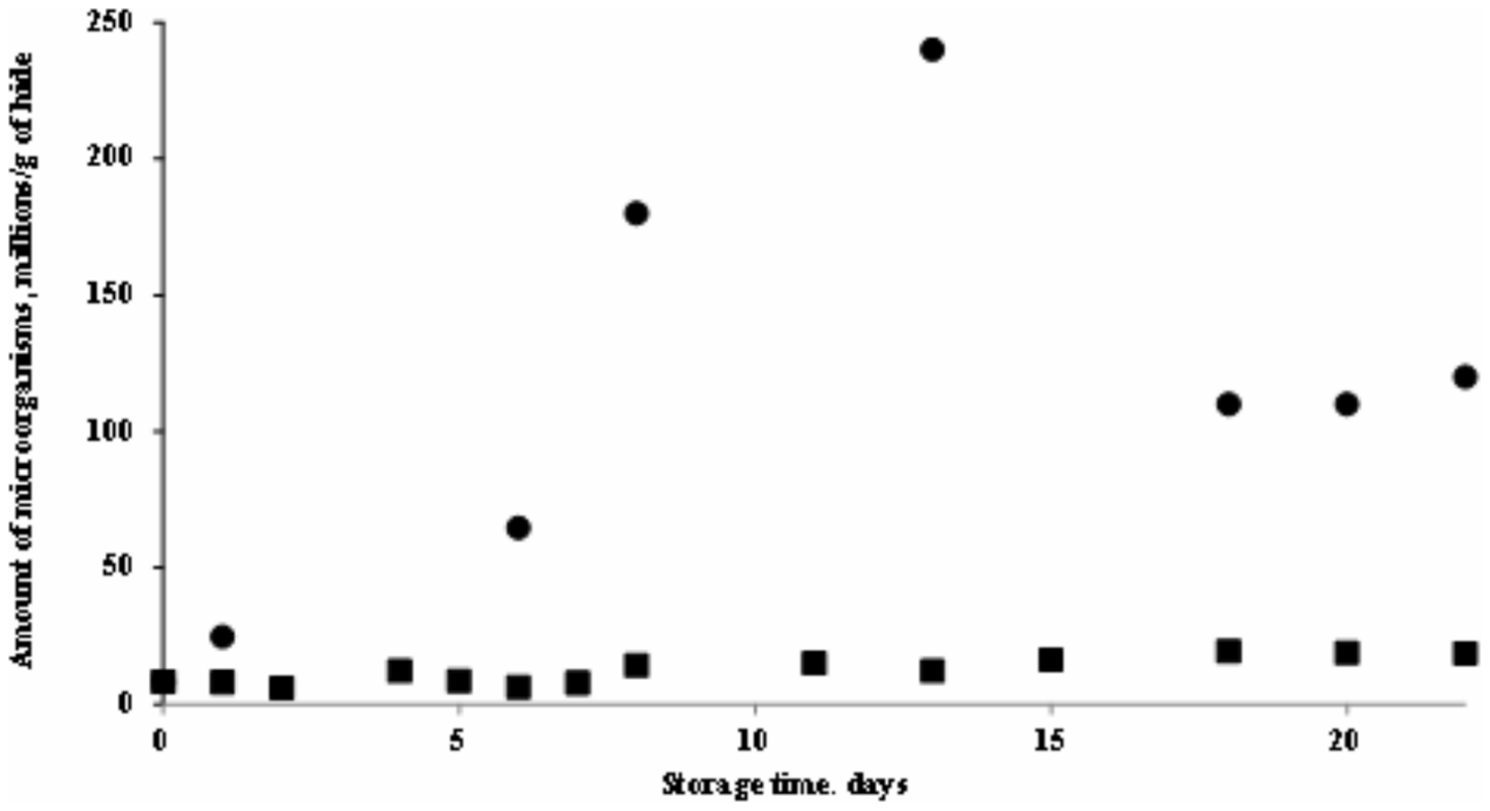
Microorganisms reproduction kinetics in hide: ▪ – vacuumed;• – not preserved.

Therefore, the first step was to determine the action of vacuum on bacterial growth. The results obtained have shown that the vacuum and low temperature block the vigorous multiplication of bacteria. During a period of 22 days, the number of microorganisms was found to increase from 8 to 18 million but did not reach the critical value (**Fig. 2**). On the other hand, bacteria present on hide slowly but act during that mentioned time. Due to this, a weakened bond of hair with derma was observable after 22 days of storage. Also, a slight odour had begun to appear. These results indicated the beginning of the slow process of decay.

A very interesting picture was obtained by observing bacterial growth on unpreserved hide stored at ambient temperature (20±2°C) (**Fig. 2**). During 2 weeks, an impetuous multiplicative process of microorganisms was observed. After that, the amount of bacteria began to decrease as it “the logarithmic dying rate phase of the bacteria” [31] had been reached.

The next step was to discover how the action of microorganisms affects derma collagen. The index, which is very sensitive to collagen structural changes, is the shrinkage temperature of hide. The results of hide shrinkage temperature determination (**Table 2**) did not show any serious changes in derma structure. For all samples (independent of the preservation method used), only a negligible decrease of shrinkage temperature was observed. This indicates that the action of microorganisms, which takes place only on the surface of the hide, does not affect the deeper layers of the hide. This remains true even when bacteria act in a very intensive manner (as was the case with unpreserved hide). Therefore, it can be concluded that changes in shrinkage temperature do not reflects a level of hide deterioration when no chemical material is used for hide preservation, and collagen, accordingly, is not affected by this material.

**Table 2:** Kinetic of hide shrinkage temperature during storage.

Hide storage duration, days	Shrinkage temperature (°C) of hide		
	not preserved	vacuumed	salted
3	63.3	64.3	64.3
6	63.7	63.7	63.4
9	61.7	62.3	62.3
13	62.3	63.3	63.0
16	62.3	62.7	63.0
20	62.0	62.0	63.0
21	61.3	62.3	62.0

Note. Shrinkage temperature of fresh hide was 64.7°C.

Another index which indicates a condition of preserved hide is the amount of ammonia released, which is formed when the hydrolysis of proteins begins due to the action of microorganisms. The data in **Table 3** show that the observable increase of the amount of extracted nitrogen begins only for unpreserved hide after 13 days of storage. This result confirms a proposition that hide can be stored at 4°C for 2 weeks without compromising its quality [1]. In the case of vacuumed and salted hide samples, the increment of nitrogen content was negligible.

**Table 3:** Change of nitrogen content extracted from hide.

Hide storage duration, days	Amount (g/kg of hide) of nitrogen extracted from hide		
	not preserved	vacuumed	salted
3	4.14	3.45	3.07
13	4.56	3.82	3.23
16	4.65	3.40	3.26
20	5.48	4.07	3.88
21	7.06	4.24	4.12

**Figure 3:**
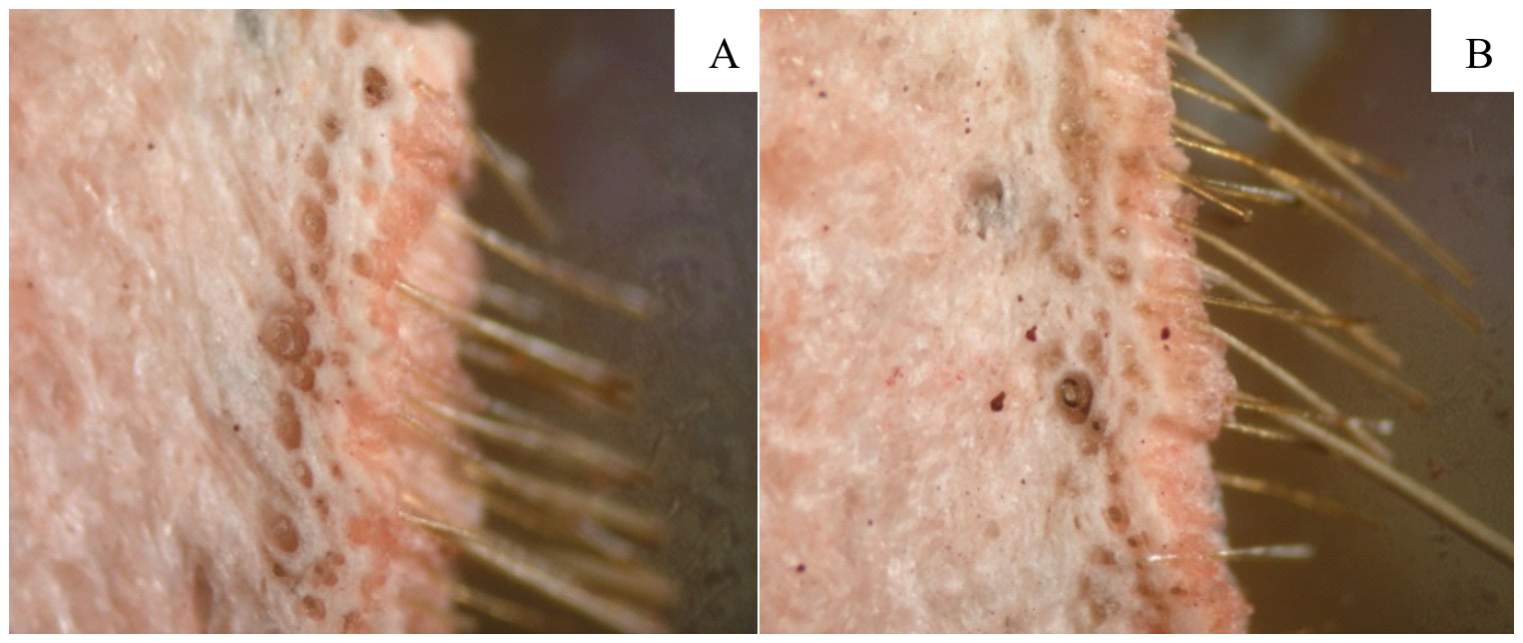
Images of vacuumed hide (magnification 40 times) stored 1 day (A) and 22 days (B).

One of initial symptoms of deterioration is a change to the exterior of the hide. As a result, a comparison of the hide samples was carried out. The optical microscopy images are presented in **Fig. 3** **.** The exploration of images allowed the conclusion to be drawn that any observable changes to the outside and inside of the hide did not occur during the 22 day storage period.

**Figure 4:**
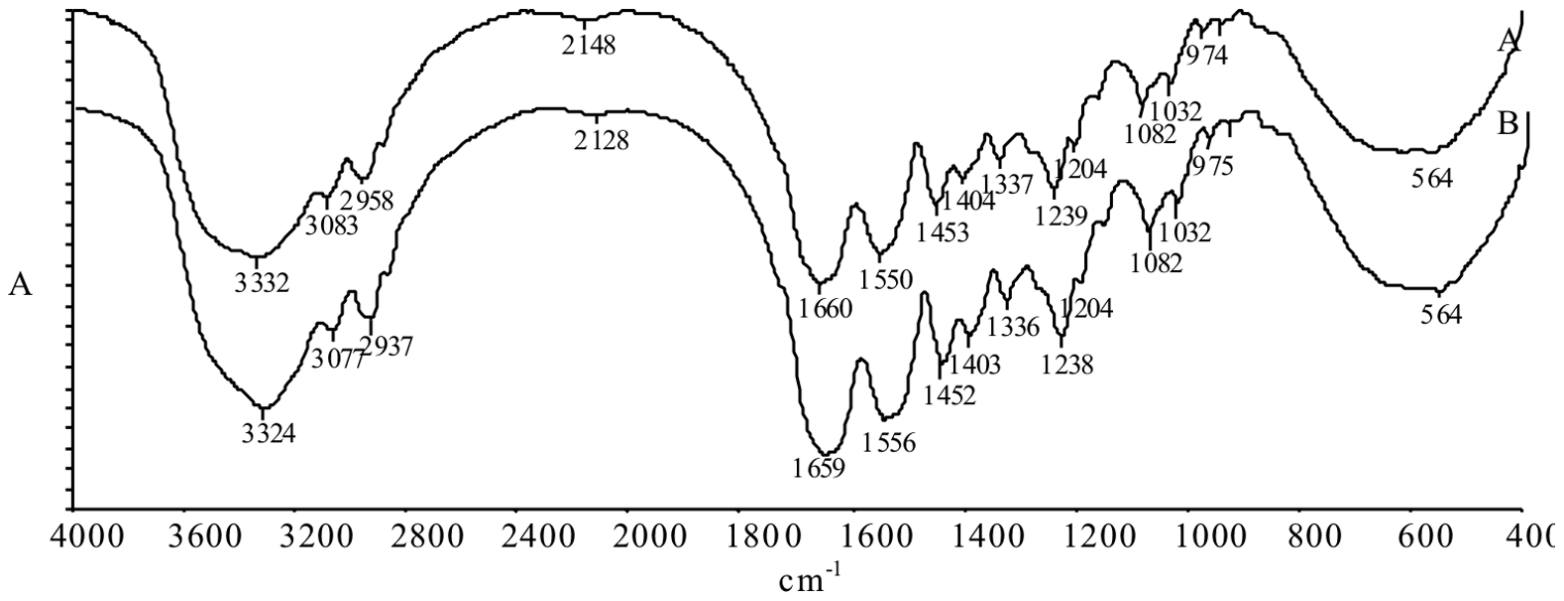
IR spectra of vacuumed hide samples stored 1 day (A) and 22 days (B).

**Figure 5:**
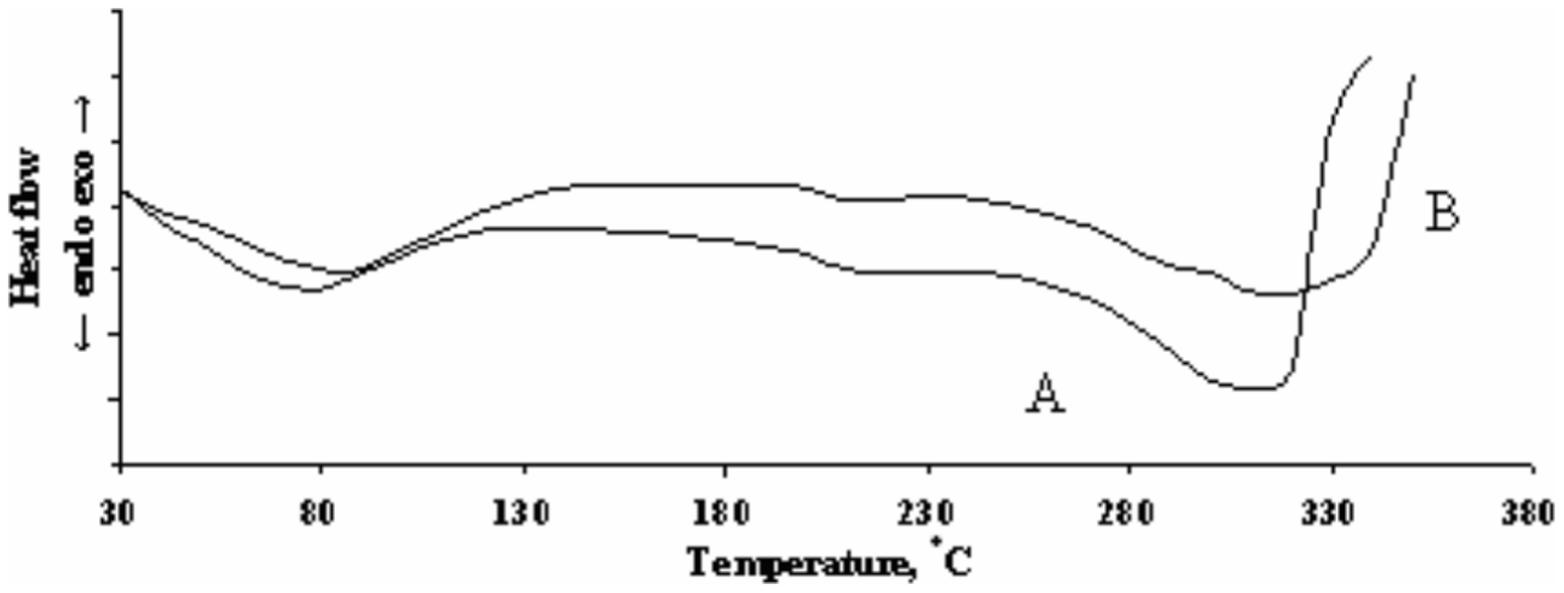
DSC curves of vacuumed hide samples stored 1 day (A) and 22 days (B).

Furthermore, possible changes of the supermolecular structure during storage under vacuum were investigated. FTIR spectroscopy (**Fig. 4** **,** **Table 4**) and DSC analysis (**Fig. 5** **,** **Table 5**) were carried out for this purpose. The structural changes reflect in the FTIR spectra as changes of individual bands in both position and intensity [31]. A comparison of the peak area values allows a conclusion to be drawn about the formation of functional groups and the degradation or formation of bonds during hide storage. The most typical bands in the spectra were chosen for evaluation of structural changes.

**Table 4:** Data of FTIR spectrum quantitative analysis.

Functional group or bond, to which the vibration is attributed	Vacuumed hide sample storage time, days			
	1		22	
	* 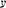 *, cm^−1^	Δ*S*	* 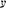 *, cm^−1^	Δ*S*
N-H; O-H	3332	17522	3325	28119
= NH; -CH_3_	2958	2230	2937	3375
= C = O “amide band I”	1660	8328	1659	10635
“amide band II”	1550	255	1556	363
–OH; R-COO–	1453	651	1452	1112
“amide band III”	1239	886	1238	1358

**Table 5:** Delimitation of thermal effects in DSC curves.

Hide storage time, days	First endothermic effect, °C			Second endothermic effect, °C			Third endothermic effect, °C		
	start	finish	Δ*t*	start	finish	Δ*t*	start	finish	Δ*t*
1	73	111	38	204	211	7	274	321	47
22	87	128	41	204	212	8	265	325	60

From studying the spectra of the hide samples (**Fig. 4**) it can be seen that no novel peaks were identified, and there was no loss of existing peaks. This indicated that any serious changes in supermolecular structure of collagen do not occur during the 22 day storage period. On the other hand, the intensity of all peaks was found to increase (**Table 4** **)**. The authors hypothesise that, owing to the action of vacuum during storage, the collagen fibres slowly become closer, making the derma becomes denser, which leads to the increased peak intensity in spectra from the hide samples that were stored for 22 days.

The results of DSC analysis are presented in **Fig. 5** and **Table 5**. It can be seen (**Fig. 5**) that DSC curves have three distinct thermal effects. The first endothermic effect can be attributed to shrinkage (denaturation) of the hide [32]. However, a more credible suggestion is the association of that effect with the removal of capillary moisture [33]. It is known [34] that the degree of linking of capillary moisture in collagen is higher when capillaries are smaller. Presumably, the closer proximity of fibres during storage under vacuum leads to a decrease of capillary dimensions, and accordingly, to an increase of the initial and final temperatures of the first endothermic effect.

Kutianin et al. related the second temperature effect to the change of the crystal phase to an amorphous state [35]. When processing a hide, the level of crystallinity becomes lower when the hide is affected stronger during processing [36]. In our case, the start and finish temperatures of the second endothermic effect coincide for both hide samples, which proves that collagen is not affected.

The third endothermic effect is related to the destruction of hide tissue. After 22 days of storage, this results in lower starting and higher finishing temperatures compared with those samples which were stored for 1 day. The lower initial temperature indicates the beginning of the deterioration of the outer layers of the hide, but due to pressed collagen fibres, the thermal effect ends at a higher temperature.

Summarising the results, it can be concluded that hide storage at 4°C under vacuum can prolong the storage duration to 3 weeks without any observable symptoms of hide tissue deterioration and without any changes to the hide collagen structure.

Of course, one or other preservation methods can be validated only by checking the quality of the leather processed from hide preserved by this method. Since leather quality depends upon the condition of the raw material, the behaviour of the hide during processing and the properties of the leather produced from such hide confirm or deny the suitability of preserved hide for the manufacture of leather. As the main protein in hide is collagen, its condition determines the quality of the leather produced. This was why the changes in collagen were initially investigated.

The processing of leather from hide samples stored for 5 and 19 days under vacuum was carried out. Such solutions were conjugated: liming and washing after liming; deliming-bating and washing after deliming-bating. These mixtures and pickling solutions were analysed with the aim of establishing the amount of collagen proteins removed. The results are presented in **Table 6**.

**Table 6:** Dependence of amount (g/1 kg of hide) of removed from hide collagen proteins on technological process.

Sort of hide	Amount of removed collagen proteins during process				Total amount during all processes
	soaking	liming	deliming-bating	pickling	
Not preserved	–	0.34	0.23	0.02	0.59
Vacuumed (stored 5 days)	–	0.30	0.22	0.02	0.54
Vacuumed (stored 21 day)	–	0.38	0.18	0.03	0.59
Salted (stored 21 day)	0.07	0.20	0.13	0.02	0.42

It is known that the amount of collagen proteins removed during liming usually varies in the range 0.2–0.5 g/kg of hide [36], [37]. When liming the vacuumed hide stored for 21 days, the amount of collagen proteins removed was higher than those removed from salted hide. Also, it somewhat exceeded the amount removed from unpreserved hide or that stored for a shorter period of time following vacuum. On the other hand, the amount of collagen proteins removed during liming was not higher than the above-mentioned value (0.5 g/kg of hide) for any of the samples tested. Comparison of the total amount of collagen proteins removed during all beamhouse processes was very close to the results obtained for vacuumed or unpreserved hide. It can be concluded, therefore, that vacuumed hide is no more affected than unpreserved hide when it is processed.

The second step was the investigation of hide properties during the chrome tannage process. Exhaustion of chromium compounds, content of Cr_2_O_3_ in leather and distribution of chromium in separate leather layers were evaluated after tanning. Data are presented in **Table 7**.

**Table 7:** Qualitative indexes of chroming process and tanned leather.

Index	Leather produced from vacuumed hide stored	
	5 days	19 days
Exhaustion of chromium compounds, %	79.9	84.8
Shrinkage temperature, °C	104	109
Cr_2_O_3_ content in leather, %	3.95	4.04
Cr_2_O_3_ content in leather’s separate layers, %		
upper	4.76	5.28
middle	3.38	3.92
lower	4.74	4.62

The comparison of chroming process indexes showed that better results were achieved when the hide samples stored for longer periods of time (19 days) were processed. In such cases, the exhaustion of chromium compounds was higher. Accordingly, the amount of Cr_2_O_3_ in this leather was also higher. This can be explained on the grounds of more intense opening up of the derma structure in the case of hide stored for a longer period prior to processing. As a result, the derma of this sample was more accessible for chromium compounds, which led to the higher chromium exhaustion and to higher Cr_2_O_3_ content in the leather.

Consequently, the higher content of Cr_2_O_3_ leads to higher shrinkage temperature, which is the main reason for the higher shrinkage temperature of the leather produced from the hide stored under vacuum for 19 days.

The distribution of Cr_2_O_3_ in both leather samples is sufficient and similar. Therefore, the longer storage time of hide has had no observable influence on this leather quality index.

Of course, the most reliable estimation of the suitability of hide preserved for longer times under vacuum for processing can only be made following industrial trials. Therefore, the hide samples preserved by vacuum and stored for 5 and 19 days were processed under industrial conditions in the “Kedainiu oda” tannery (Lithuania) into shoe upper leather. The leather samples produced were tested and qualitative indexes were determined (**Table 8**).

**Table 8:** Chemical and physical properties of leather produced under industrial conditions from preserved by vacuum hides.

Index	Leather produced from hide stored in vacuum	
	5 days	19 days
Tensile strength, N/mm^2^	18.8	19.7
Relative elongation when the strain of 10 N/mm^2^ is reached, %	54.5	47.3
Relative elongation at break, %	78.7	70.3
Strain when grain breaks, N/mm^2^	15.6	18.1
Cr_2_O_3_ content, %	3.15	3.34
Matter soluble in dichloromethane, %	4.93	5.04
Volatile matter, %	15.3	15.3
Shrinkage temperature, °C	104	108

The values obtained for the main properties showed that, in both cases, high quality leather was produced. The strength properties entirely satisfied the requirements for this type of leather. Industrial trials confirmed the results obtained under laboratory conditions: after longer storage periods, hide bound a higher amount of chromium compounds during chroming and had higher thermostability compared with hide stored for shorter periods. The content of soluble matter in dichloromethane was sufficient and similar for both leathers.

## Conclusion

Vacuum can be used for the short-term preservation of hide. It allows prolongation of the storage time without hide tissue putrefaction of up to 21 days when the storage temperature is 4°C. The number of microorganisms during that time does not reach the level of 20×10^6^ per g of hide. The microorganisms for all storage times are active but this activity is weak and has no observable influence on the quality of the hide. The decrease in hide shrinkage temperature is negligible, which shows that breakage of intermolecular bonds does not occur during the mentioned storage times.

FTIR spectroscopy and DSC analysis did not show any serious structural changes, which can influence the quality of the leather produced from vacuumed hide in the future. Negligible changes in FTIR spectrum and in DSC curves of the hide samples stored for three weeks are indicative of pressing of the hide tissue and closing up of the collagen fibres.

The duration of hide storage under vacuum at low temperature has an influence on the level of collagen proteins removed during processing of hide into wet blue. The amount of removed collagen proteins following 21 days of storage during liming, deliming-bating and pickling processes was about 1.5-times higher than that from salted hide. Despite this, leather produced from hide stored for 19 days bound more chromium compounds and had a higher shrinkage temperature. Properties of the leather produced under industrial conditions also conformed to the requirements of shoe upper leather.

The results obtained allow the conclusion that hide under vacuum at low temperature can be stored for 21 days, and this does not lower the quality of the leather produced.
